# The Deubiquitinase USP22‐Stabilized COL17A1 Promotes Lung Adenocarcinoma Progression

**DOI:** 10.1111/crj.13824

**Published:** 2024-08-14

**Authors:** Guangxi Chen, Dandan Du, Haihua Wang, Huifeng Li

**Affiliations:** ^1^ Department of General Medicine, Jiujiang City Key Laboratory of Cell Therapy Jiujiang NO.1 People's Hospital Jiujiang China; ^2^ Department of Jiulong Community Health Service Centre, Jiujiang City Key Laboratory of Cell Therapy Jiujiang NO.1 People's Hospital Jiujiang China; ^3^ Department of Chronic Hepatology Jiujiang Third People's Hospital Jiujiang China; ^4^ Department of Respiratory and Critical Care Medicine Jiujiang Third People's Hospital Jiujiang China

**Keywords:** COL17A1, deubiquitination, ferroptosis, lung adenocarcinoma, USP22

## Abstract

**Background:**

Lung adenocarcinoma (LUAD) is a highly aggressive and rapidly fatal malignancy worldwide. Collagen XVII (COL17A1) has been implicated in various protumorigenic processes. However, the functions and mechanisms of COL17A1 in LUAD progression still remain elusive.

**Methods:**

COL17A1 and ubiquitin‐specific protease 22 (USP22) mRNA analysis was performed by quantitative PCR, and their protein levels were detected by immunoblotting and immunohistochemistry. The functional influence was evaluated by determining cell viability, proliferation, apoptosis, invasion, migration, and ferroptosis in vitro, as well as xenograft growth in vivo. Co‐immunoprecipitation (Co‐IP) and IP experiments were used to examine the USP22/COL17A1 interaction and COL17A1 deubiquitination. Cycloheximide treatment was used to analyze COL17A1 protein stability.

**Results:**

COL17A1 and USP22 were upregulated in human LUAD tissues and cell lines. Functionally, COL17A1 knockdown acted for the suppression of LUAD cell growth, invasion, and migration as well as promotion of cell apoptosis and ferroptosis in vitro. COL17A1 knockdown could diminish the tumorigenicity of LUAD cells in vivo. Mechanistically, USP22 stabilized and upregulated COL17A1 by enhancing the deubiquitination of COL17A1. Additionally, reexpression of COL17A1 could reverse USP22 silencing‐induced phenotype changes of LUAD cells in vitro.

**Conclusion:**

Our findings demonstrate that USP22‐stabilized COL17A1 possesses oncogenic activity in LUAD. We propose that USP22 and COL17A1 would be potential targets for the establishment of therapeutic approaches against LUAD.

## Graphical abstract

In lung adenocarcinoma cells, the deubiquitinase USP22 stabilizes COL17A1 protein by enhancing COL17A1 deubiquitination to upregulate COL17A1 and thus promotes cell growth, invasion, and migration and suppresses cell apoptosis and ferroptosis, leading to this disease progression.

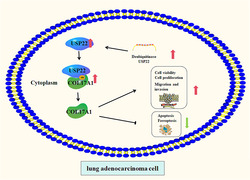

## 1 Introduction

Lung adenocarcinoma (LUAD) is the prevalent histological subtype of lung cancer and is known to be highly aggressive and often fatal in East Asians. Despite achievements in understanding of the pathogenesis of LUAD and improvements in therapeutic regimens, overall survival rates remain poor [1]. Therefore, there is an urgent need to exploit novel approaches against LUAD. Ferroptosis, a distinct type of cell death, has attracted significant interest in LUAD research due to its crucial roles in the development of this disease [2, 3]. Resistance to ferroptosis is associated with enhanced progression of LUAD, making enhancing ferroptosis sensitivity a key principle in ferroptosis‐based treatment [2]. Uncovering the molecular pathways governing ferroptosis and LUAD progression would contribute to the development of innovative and more efficient targeted therapies for LUAD.

The single‐transmembrane protein collagen XVII (COL17A1) is a component of hemidesmosomes that connect basal cells and the underlying basement membrane. A previous study reported in *Current Biology* highlights the crucial role of COL17A1 in the formation of multilayered epithelial structures, an important event in the early stages of tumorigenesis, suggesting the essential implication of COL17A1 in cancer development [4]. Computational algorithm analyses have shown that COL17A1 is closely related to the tumor microenvironment, prognosis, and chemosensitivity in pancancer by acting as an immune‐related factor [5]. Moreover, dysregulation of COL17A1 has been demonstrated to contribute to protumorigenic processes, with COL17A1 being emerged as either a cancer driver or antitumor factor. As an example, in colorectal cancer, upregulated COL17A1 promotes disease progression by elevating the expression of immunosuppressive cytokines [6]. Conversely, COL17A1 is capable of blunting breast cancer cell growth by blocking the AKT/mTOR pathway [7]. In pancreatic cancer, COL17A1's impact on in vivo tumor growth varies depending on the tumor microenvironment [8]. In LUAD, hypomethylation of the *COL17A1* promoter is observed [9], implying the elevated expression of the COL17A1 protein in this cancer. However, it still remains elusive whether and how COL17A1 has been implicated in LUAD progression.

The ubiquitination/deubiquitination system controls protein fate and thus strongly regulates a broad spectrum of important cellular processes [10]. Protein modulation by this system plays a significant role in the pathogenesis of lung cancer [11]. Moreover, numerous deubiquitinases, such as USP9X, USP7, and USP5, can induce the deubiquitination of certain oncoproteins, thereby driving lung carcinogenesis [12, 13, 14]. However, it has not been defined whether the deubiquitination regulation participates in COL17A1 dysregulation during LUAD progression.

In the current work, we examined COL17A1 expression in human LUAD and then manipulated COL17A1 to elucidate its exact functions in LUAD progression using two LUAD cell lines. We further performed mechanism analyses to uncover a deubiquitination‐related molecular basis for driving COL17A1 dysregulation. This study may provide encouraging ways for LUAD treatment.

## 2 Materials and Methods

### 2.1 Bioinformatics

We utilized the TIMER2.0 website (http://timer.cistrome.org/) to observe the expression of COL17A1 in pancancer versus the matched normal tissues and the GEPIA Version 2 (http://gepia2.cancer‐pku.cn/#index) and UALCAN (https://ualcan.path.uab.edu/) databases to analyze COL17A1 expression in LUAD tumors. To search for genes related to COL17A1 in LUAD, we utilized the LinkedOmics database (https://www.linkedomics.org/login.php), which was also used to plot the ubiquitin‐specific protease 22 (USP22)/COL17A1 correlation in LUAD. We defined the genes as COL17A1‐associated genes based on the criteria of Pearson's correlation coefficient > 0.5 and *p* < 0.05. The COL17A1‐associating deubiquitinases were found using the UbiBrowser2.0 database (http://ubibrowser.bio‐it.cn/ubibrowser_v3/).

### 2.2 Collection of Human Clinical Specimens

With informed consent signed by all participants, we collected pathologically staged LUAD tumors (*n* = 45) and paraneoplastic normal lung samples (*n* = 45) from 45 LUAD sufferers from Jiujiang NO.1 People's Hospital. None of the patients received systemic or local therapy before surgery, and all had detailed clinical information. The clinicopathologic features of these patients are presented in Table 1. These clinical specimens were stored in a Thermo Fisher Scientific freezer (Thermo Fisher Scientific, Saint‐Aubin, France) at −80°C until use for the expression analysis of COL17A1 and USP22. Human studies were conducted following the approved protocols through the Jiujiang NO.1 People's Hospital Ethics Committee.

**TABLE 1:** Correlation of COL17A1 expression with the clinicopathologic features of LUAD patients (*n* = 45).

Parameters	*N* = 45	COL17A1 expression		*p* value
		High *N* = 23	Low *N* = 22	
Age (years)				
<60	29	17	12	0.1749
≥60	16	6	10	
Sex				
Male	19	12	7	0.1670
Female	26	11	15	
History of smoking				
Have	31	18	13	0.1650
No	14	5	9	
Differentiation				
Low	17	12	5	0.0417^*^
Middle and high	28	11	17	
TNM stage				
I + II	11	9	2	0.0191^*^
III	33	14	20	
Lymph node metastasis				
No	17	11	6	0.1901
Yes	27	12	15	

^*^ *p* < 0.05, statistically significant.

### 2.3 mRNA Analysis in Human Specimens by Quantitative PCR

Under the use of Isogen as described by the vendor (Nippon Gene, Tokyo, Japan), we prepared total RNA from human clinical specimens. cDNA generation was achieved with 1 μg RNA using random hexamers and the QuantiTect Reverse Transcription Kit (Qiagen, Crawley, United Kingdom). The diluted cDNA was then used for SYBR‐based quantitative PCR (Servicebio, Wuhan, China) with primers specific for COL17A1 (5′‐CCTGGACAAAATTGGGCTGC‐3′‐sense and 5′‐CCTGGACTTCCCATGTCACC‐3′‐antisense) or USP22 (5′‐GGACAACTGGAAGCAGAACC‐3′‐sense and 5′‐AGATACAGGACTTGGCCTTGC‐3′‐antisense). Relative COL17A1 and USP22 mRNA expression was quantified by the 2^‐ΔΔCt^ method after normalization to the amount of β‐actin (5′‐GAGAAAATCTGGCACCACACC‐3′‐sense and 5′‐GGATAGCACAGCCTGGATAGCAA‐3′‐antisense).

### 2.4 Cell Lines

We purchased human A549 (Cat No. #CL‐0016), H1975 (Cat No. #CL‐0298), H1299 (Cat No. #CL‐0165), and H23 (Cat No. #CL‐0397) LUAD cells from Procell (Wuhan, China), as well as normal pulmonary epithelial BEAS‐2B cells (Cat No. #C6106) from Beyotime (Shanghai, China). We propagated all cell lines in complete RPMI‐1640 media (RPMI‐1640, 10% fetal calf serum, 100 μg/mL streptomycin, and 100 IU/mL penicillin) (all from Life Technologies, Lucerne, Switzerland) with the exception of A549, which was propagated using Ham's F‐12K (Biochrom KG, Berlin, Germany).

### 2.5 Constructs, Transfection, and Stable Cell Line Generation

We purchased COL17A1‐specific (sh‐COL17A1) and USP22‐specific (sh‐USP22) shRNAs, as well as a nontarget shRNA control (sh‐Ctrl), from Fenghui Biotechnology (Changsha, China). The sh‐COL17A1 lentiviral particles and sh‐Ctrl control virus were constructed by WZ Biosciences (Jinan, China). We obtained the pCDNA3.1‐V5‐FLAG‐USP22 (oe‐USP22) to express USP22 from Miaoling (Wuhan, China).

The in vitro knockdown (KD) and/or reexpression was achieved following the producer's suggestions (Baidai, Changzhou, China). In brief, we seeded H1975 and A549 LUAD cells in 12‐well (5 × 10^4^ cells/well) or 96‐well (1 × 10^3^ cells/well) culture plates 12 h prior to transient transfection. These cells were subjected to transfection with RFect Plasmid Transfection Reagent mixed with sh‐Ctrl, sh‐COL17A1, sh‐USP22, or sh‐COL17A1 + oe‐USP22. We harvested transfected cells at 48 h post transfection for the subsequent assays described below. The in vivo COL17A1 KD was achieved by generating a stable A549 cell line. Briefly, we seeded A549 LUAD cells in 10‐cm culture dishes 1 day prior to virus infection (sh‐COL17A1 or sh‐Ctrl) in media plus polybrene (4 μg/mL, Solarbio, Beijing, China). For the selection of a stable cell line, we applied puromycin (Macklin, Shanghai, China) in a concentration of 2 μg/mL after 72 h of infection.

### 2.6 Animal Experiments and Immunohistochemistry

Under approval from Jiujiang NO.1 People's Hospital Animal Ethics and Experimentation Committee, we carried out all animal work with 12 female Balb/c‐nu mice (6–8 weeks, Vital River Laboratory Animal Technology, Beijing, China), which were randomly grouped into designated groups: sh‐COL17A1 (*n* = 6) and sh‐Ctrl (*n* = 6). We established xenograft tumors by subcutaneous injection of sh‐COL17A1‐infected or sh‐Ctrl‐transduced A549 cells (5 × 10^6^ cells/mouse). We evaluated xenograft growth by periodically analyzing their volume using the (shortest diameter)^2^ × (longest diameter)/2 method. After 30 days, xenograft tumors were weighed, fixed in 4% formaldehyde, and subjected to immunohistochemistry staining as described elsewhere [15], using anti‐Ki67 (rabbit polyclonal, Cat No. #ab15580, Abcam, Cambridge, United Kingdom; 1–300), anti‐COL17A1 (rabbit monoclonal, Cat No. #ab184996, Abcam; 1–100), or anti‐GPX4 (mouse monoclonal, Cat No. #67763‐1‐Ig, Proteintech, Wuhan, China; 1–2000) antibody.

### 2.7 Protein Analysis by Immunoblotting

We performed protein preparation using the lysis buffer consisting of 20 mM Tris (pH = 7.2), 3 mM EDTA, 250 mM NaCl, 0.5% NP‐40, 1% Triton‐X, and a protease inhibitor cocktail (Life Technologies). Immunoblotting was conducted as previously described [15, 16] with antibodies including anti‐COL17A1 (rabbit monoclonal, Cat No. #ab184996, Abcam; 1–1000), anti‐USP22 (rabbit polyclonal, Cat No. #55110‐1‐AP, Proteintech; 1–5000), anti‐ACSL4 (rabbit monoclonal, Cat No. #ab155282, Abcam; 1–30 000), anti‐SLC7A11 (rabbit monoclonal, Cat No. #ab307601, Abcam; 1–1000), anti‐GPX4 (mouse monoclonal, Cat No. #67763‐1‐Ig, Proteintech; 1–4000), anti‐ubiquitin (anti‐UB, rabbit polyclonal, Cat No. #10201‐2‐AP, Proteintech; 1–5000), and anti‐β‐actin loading buffer (rabbit polyclonal, Cat No. #ab8227, Abcam; 1–4000).

### 2.8 Cell Viability, Proliferation, and Apoptosis Assays

H1975 and A549 LUAD cells grown in 96‐well culture plates were subjected to the appropriate transfection and checked for cell viability by the CCK‐8 assay and for proliferating ability by the EdU assay. For viability analysis, CCK‐8 reagent was applied as suggested by the producer (Servicebio), and the viable cells were evaluated by reading the absorbance at 450 nm. For proliferation analysis, the Yefluor 488 EdU Imaging Kit was utilized as per the vendor's instructions (Yeasen, Shanghai, China), and the EdU‐positive cells (showing a green fluorescence) were expressed as a percentage of total cells (showing a blue fluorescence).

The apoptotic cells were analyzed by flow cytometry on a FACSCalibur (BD Biosciences, Heidelberg, Germany). H1975 and A549 LUAD cells after the appropriate transfection were stained with Annexin V and PI using the Annexin V‐FITC Apoptosis Assay Kit (Beyotime). Data were acquired within 1 h after staining.

### 2.9 Transwell In Vitro Migration and Invasion Assays

We resuspended H1975 and A549 LUAD cells after the appropriate transfection in serum‐free RPMI‐1640 and plated them in a Matrigel invasion chamber with a PET membrane with an 8 μm pore (for the invasion experiment, Corning BioCoat, Lindfield, New South Wales, Australia) or homogenous 24‐transwell (for the migration experiment, Corning BioCoat). The chamber was then placed in matched 24‐well culture plates, which were filled with 700 μL of complete RPMI‐1640 media. After 24 h of culture and crystal violet (1%) staining, an Olympus X‐71 microscope (Olympus, Tokyo, Japan) was applied for imaging, and the ImageJ software (NIH, Bethesda, Maryland, United States) was used to analyze the invaded or migratory cells at five random fields.

### 2.10 ROS Detection

For ROS detection in H1975 and A549 LUAD cells after the appropriate transfection, we applied the DCFH‐DA‐basic ROS Assay Kit as recommended by the supplier (Beyotime). The Olympus fluorescence microscope was used for imaging.

### 2.11 Determination of MDA, GSH, and Fe^2+^

We harvested H1975 and A549 LUAD cells after the appropriate transfection to analyzed their MDA, GSH, and Fe^2+^ contents, in accordance with the accompanying protocols, with a MDA Assay Kit (Abcam), a GSH Assay Kit (Enzyme‐linked Biotechnology, Shanghai, China), and a Ferrous Iron Colorimetric Assay Kit (Elabscience, Wuhan, China), respectively.

### 2.12 Co‐Immunoprecipitation (Co‐IP) and IP Experiments

For IP experiments, A549, H1975, and sh‐USP22‐ or sh‐Ctrl‐transfected A549 LUAD cells were lysed in the lysis buffer described in immunoblotting analysis. Under rotating conditions, cell extracts (1 mg) were incubated (overnight; 4°C) with 0.5 μg anti‐COL17A1 (rabbit monoclonal, Cat No. #ab184996, Abcam) or anti‐USP22 (rabbit polyclonal, Cat No. #55110‐1‐AP, Proteintech) antibody and prewashed Protein A Dynabeads (Life Technologies). We subsequently collected the immunoprecipitates for the enrichment analyses of COL17A1, USP22, or ubiquitinated COL17A1 by immunoblotting.

### 2.13 COL17A1 Stability Assay

To examine the impact of USP22 on COL17A1 protein stabilization, we treated sh‐USP22‐ or sh‐Ctrl‐transfected H1975 and A549 LUAD cells with 20 ng/mL of cycloheximide (Selleck, Shanghai, China). At 0, 3, 6, and 12 h post treatment, we measured COL17A1 protein levels by immunoblotting.

### 2.14 Data Analysis

Except as indicated in figure legends, all experiments included at least three independent samples. All results were expressed as the mean ± SD. We evaluated the statistical difference between groups using a one‐way or two‐way ANOVA or a two‐tailed *t*‐test. A p value < 0.05 was of statistical significance.

## 3 Results

### 3.1 Identification of COL17A1 as an Upregulated Factor in Human LUAD

**FIGURE 1:**
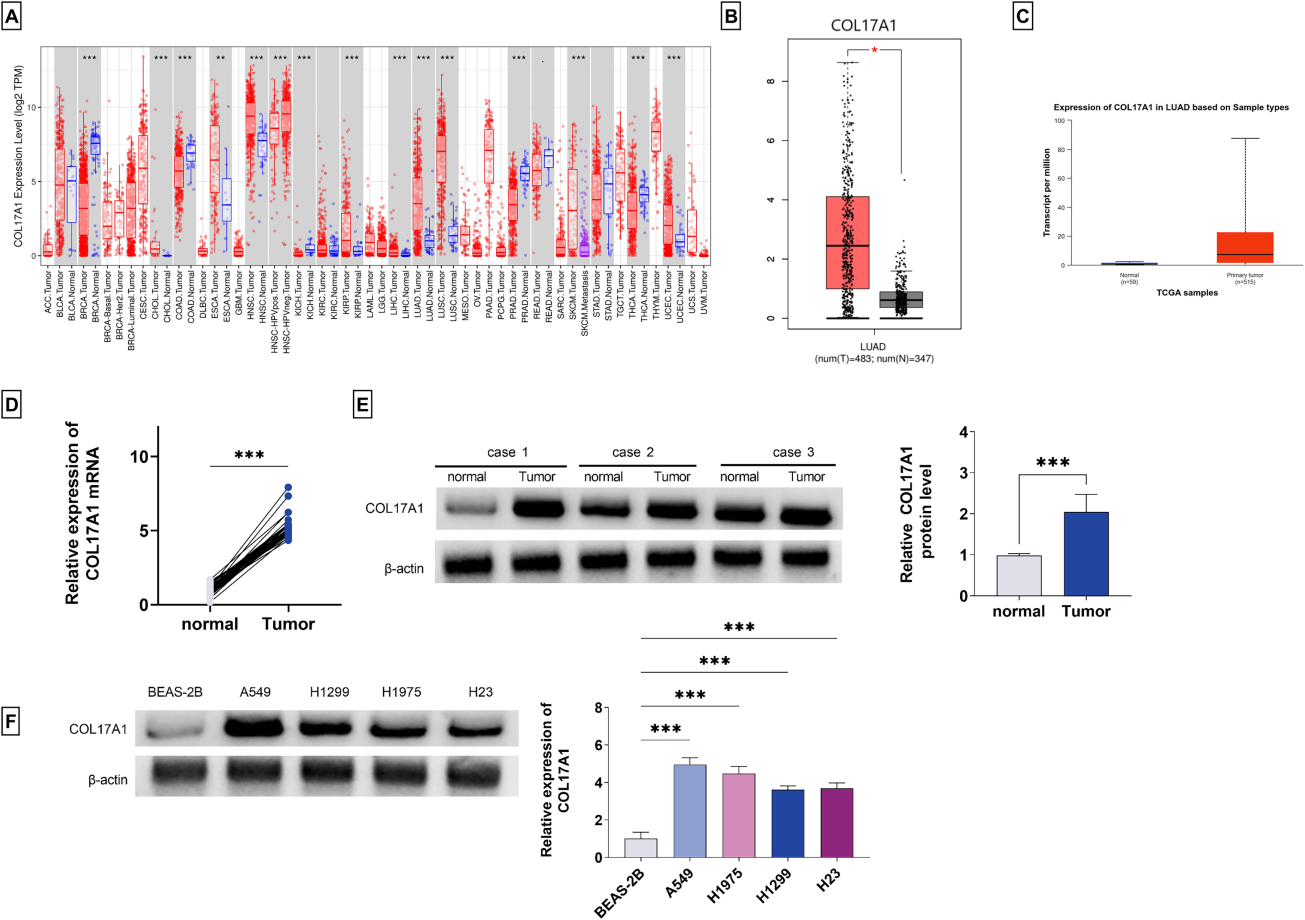
Upregulation of COL17A1 in human LUAD tumors and cells. (A) Analysis of differential expression of COL17A1 in pancancer by the TIMER2.0 website. (B, C) Analysis of COL17A1 expression in human LUAD by GEPIA Version 2 and UALCAN databases. (D) Quantitative PCR for COL17A1 mRNA in a cohort of LUAD tumors (*n* = 45) as well as matched normal lung tissues (*n* = 45). (E) Difference of COL17A1 protein levels between LUAD tumors (*n* = 3) with matched normal lung tissues (*n* = 3) detected by immunoblot analysis. (F) COL17A1 protein levels in four human LUAD cell lines (A549, H1975, H1299, and H23) as well as human normal BEAS‐2B cells detected by immunoblot analysis. ***p < 0.001.

COL17A1 has established critical roles in neoplastic transformation and cancer progression [4, 17]. However, the precise action of COL17A1 in LUAD pathogenesis is not well studied. Using the TIMER2.0 website for systematical analysis of COL17A1 in human cancer, we observed its differential expression in tumors versus matched normal tissues (Figure 1A), suggesting the dysregulation of COL17A1 in pancancer. Analysis of the TIMER2.0 website also showed the upregulation of COL17A1 in LUAD tumors compared with normal lung epithelial tissues (Figure 1A), which was also confirmed by the GEPIA Version 2 and UALCAN databases (Figure 1B,C). To confirm the COL17A1 upregulation in human LUAD, we used quantitative PCR analysis to identify COL17A1 expression in a cohort of LUAD tumors as well as matched normal lung tissues and found that COL17A1 mRNA levels were elevated in LUAD tumors compared with their normal counterparts (Figure 1D). Utilizing the commercially available antibody to human COL17A1, we confirmed the high expression of the COL17A1 protein in LUAD tumors (Figure 1E). The upregulated expression of the COL17A1 protein was also confirmed in four human LUAD cell lines (A549, H1975, H1299, and H23) compared to human normal BEAS‐2B cells (Figure 1F). Additionally, COL17A1 expression was significantly related to tumor differentiation and TNM stage (Table 1). Because of the more significant upregulation of COL17A1 in A549 and H1975 cells (Figure 1F), we used the two LUAD cell lines for the subsequent investigation.

### 3.2 COL17A1 KD In Vitro Acts for Suppression of LUAD Cell Growth, Invasion, and Migration and Promotion of Cell Apoptosis

**FIGURE 2:**
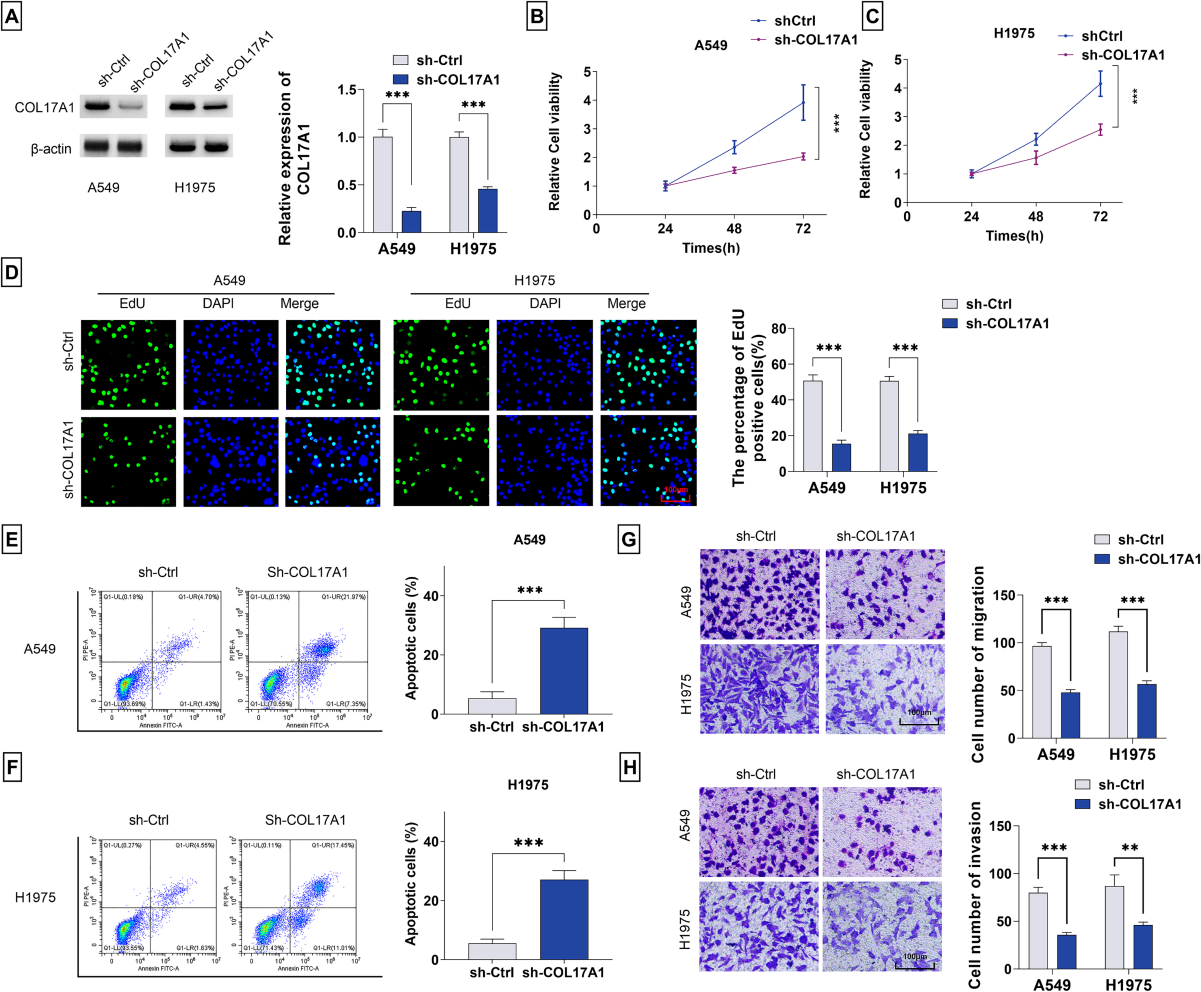
Roles of COL17A1 in growth, apoptosis, invasiveness, and motility ability of A549 and H1975 LUAD cells. (A) COL17A1 protein levels in sh‐COL17A1‐ or sh‐Ctrl‐transfected A549 and H1975 LUAD cells detected by immunoblot analysis. (B,C) Viability of sh‐COL17A1‐ or sh‐Ctrl‐transfected A549 and H1975 LUAD cells measured by CCK‐8 assay. (D) Proliferation of sh‐COL17A1‐ or sh‐Ctrl‐transfected A549 and H1975 LUAD cells assessed by EdU assay. (E,F) Apoptosis of sh‐COL17A1‐ or sh‐Ctrl‐transfected A549 and H1975 LUAD cells evaluated by flow cytometry assay. Migratory (G) and invaded (H) capacities of sh‐COL17A1‐ or sh‐Ctrl‐transfected A549 and H1975 LUAD cells measured by transwell assay. **p < 0.01, ***p < 0.001.

The above data demonstrate the upregulation of COL17A1 in human LUAD. Accordingly, we wanted to ask about the functional activity of COL17A1 in A549 and H1975 LUAD cells. To elucidate the influence of COL17A1 on in vitro cell phenotypes, we used a COL17A1‐specific shRNA (sh‐COL17A1) to deplete COL17A1 expression in A549 and H1975 LUAD cells. Immunoblot analysis confirmed that sh‐COL17A1 transfection induced a significant KD effect (Figure 2A). COL17A1 KD caused by sh‐COL17A1 led to a considerable suppressive impact on in vitro viability and proliferation of A549 and H1975 LUAD cells (Figure 2B–D). Conversely, COL17A1 KD resulted in a clear facilitation of cell apoptosis, as revealed by the flow cytometry assay (Figure 2E,F). In addition, through transwell analysis, we found that COL17A1 KD induced a striking reduction in the migratory and invaded capacities of A549 and H1975 LUAD cells (Figure 2G,H).

### 3.3 COL17A1 KD In Vitro Acts for Enhancement of LUAD Cell Ferroptosis

**FIGURE 3:**
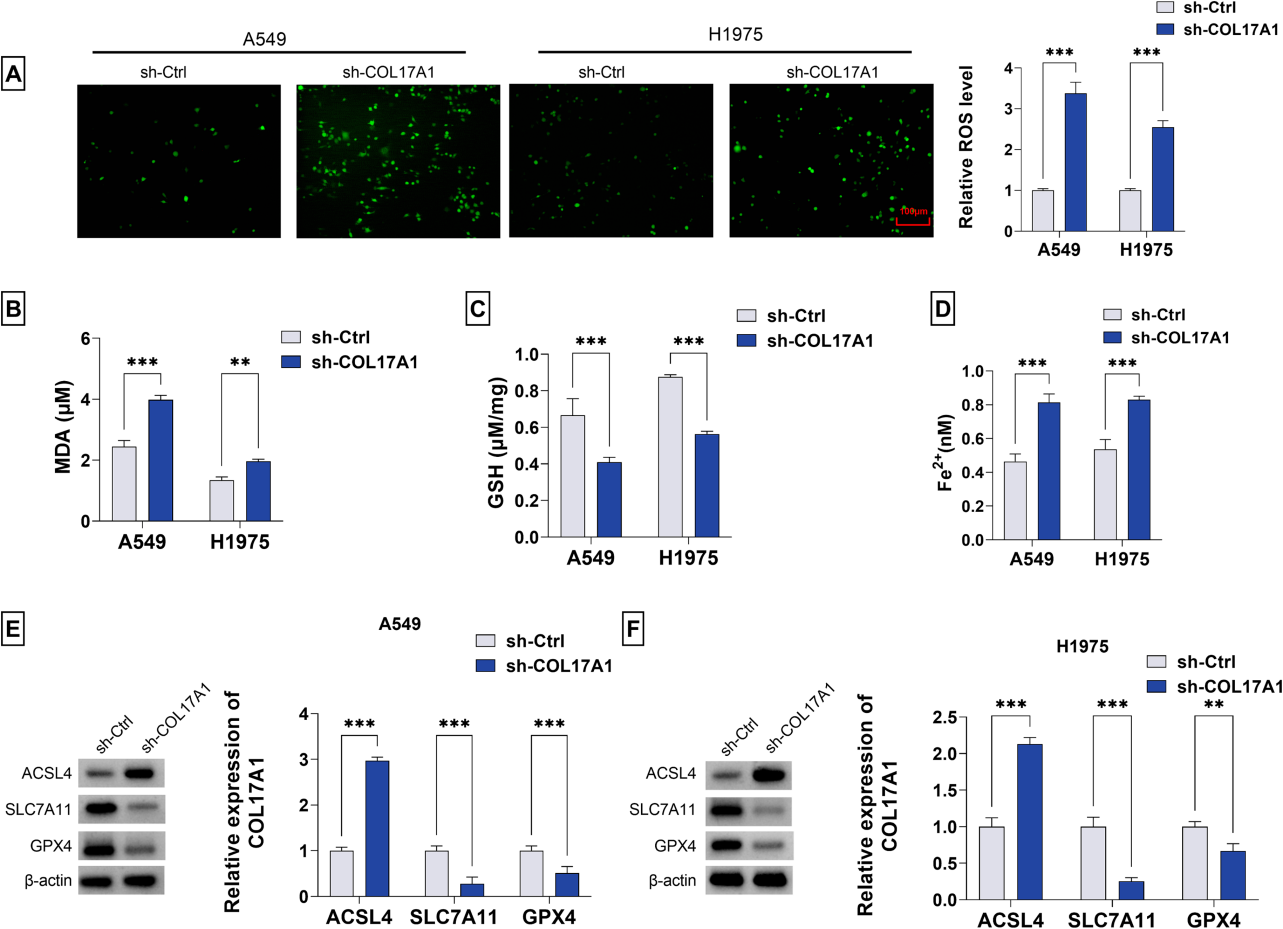
The influence of COL17A1 on ferroptosis of A549 and H1975 LUAD cells. (A) Relative ROS content of sh‐COL17A1‐ or sh‐Ctrl‐transfected A549 and H1975 LUAD cells measured by fluorescence assay. The levels of MDA (B), GSH (C), and Fe^2+^ (D) in sh‐COL17A1‐ or sh‐Ctrl‐transfected A549 and H1975 LUAD cells detected by using the relevant assay kit. (E,F) The protein levels of ACSL4, SLC7A11, and GPX4 in sh‐COL17A1‐ or sh‐Ctrl‐transfected A549 and H1975 LUAD cells assessed by immunoblot assay. **p < 0.01, ***p < 0.001.

Ferroptosis, the unique form of cell death, has garnered immense attention in lung tumorigenic process and cancer therapy [18]. Because COL17A1 has been unveiled to be related to cancer cell ferroptosis [4], we then focused on the exact action of COL17A1 in ferroptosis of A549 and H1975 LUAD cells. The accumulation and abundance of lipid ROS can induce enhanced ferroptosis [19]. Using DCFH‐DA fluorescence probes, we validated that COL17A1 KD caused by sh‐COL17A1 led to the augmentation of ROS content in A549 and H1975 LUAD cells, as illustrated by the fluorescence assay (Figure 3A). Abundance of MDA and Fe^2+^ as well as the deficiency of GSH can lead to the occurrence of ferroptosis [19, 20]. In contrast, COL17A1 KD cells exhibited increased levels of MDA and Fe^2+^ and reduced GSH content (Figure 3B–D). Moreover, immunoblot analyses revealed the upregulated expression of the ferroptosis driver ACSL4 and decreased levels of the ferroptosis inhibitors SLC7A11 and GPX4 in cells with COL17A1 KD (Figure 3E,F). In sum, these results demonstrate that COL17A1 reduction enhances ferroptosis of LUAD cells.

### 3.4 COL17A1 KD Diminishes the In Vivo Tumorigenicity of A549 LUAD Cells

**FIGURE 4:**
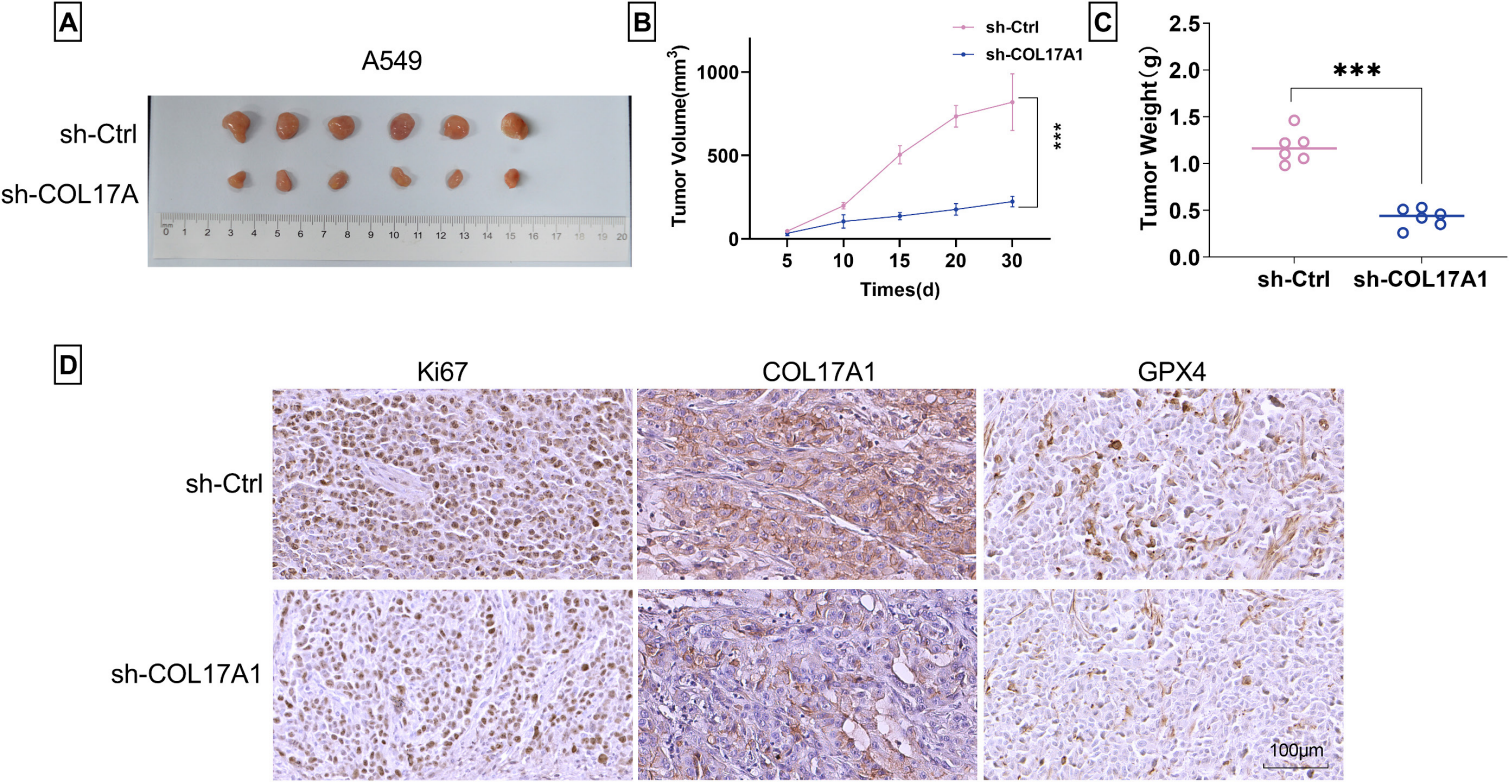
Effect of COL17A1 on the in vivo tumorigenicity of A549 LUAD cells. Images (A), growth curves (B), and average weight (C) of xenograft tumors formed by sh‐COL17A1‐ or sh‐Ctrl‐transduced A549 LUAD cells (*n* = 6). (D) Evaluation of the Ki67‐positive cells and COL17A1 and GPX4 levels in xenograft tumors formed by sh‐COL17A1‐ or sh‐Ctrl‐transduced A549 LUAD cells by immunohistochemistry assay. ***p < 0.001.

Having demonstrated the induction of COL17A1 silencing on the in vitro growth disadvantage, we further elucidated the function of COL17A1 in LUAD cells using in vivo assays. Upon transduction of the sh‐COL17A1 lentivirus in the xenograft models, tumor growth was significantly blunted, as evidenced by the decreased volume and average weight of sh‐COL17A1 xenograft tumors (Figure 4A–C). In support to this, we also evaluated the cells stained for the proliferating marker Ki67 in these xenograft models. Immunohistochemistry analysis showed fewer cells stained for Ki67 staining in sh‐COL17A1 xenograft tumors (Figure 4D). Immunohistochemistry analysis also confirmed the reduction of COL17A1 expression in sh‐COL17A1 xenograft tumors (Figure 4D). In addition, the reduced expression of the ferroptosis suppressor GPX4 was observed in sh‐COL17A1 xenograft tumors (Figure 4D).

### 3.5 USP22 Promotes the Deubiquitination of COL17A1 to Stabilize COL17A1

**FIGURE 5:**
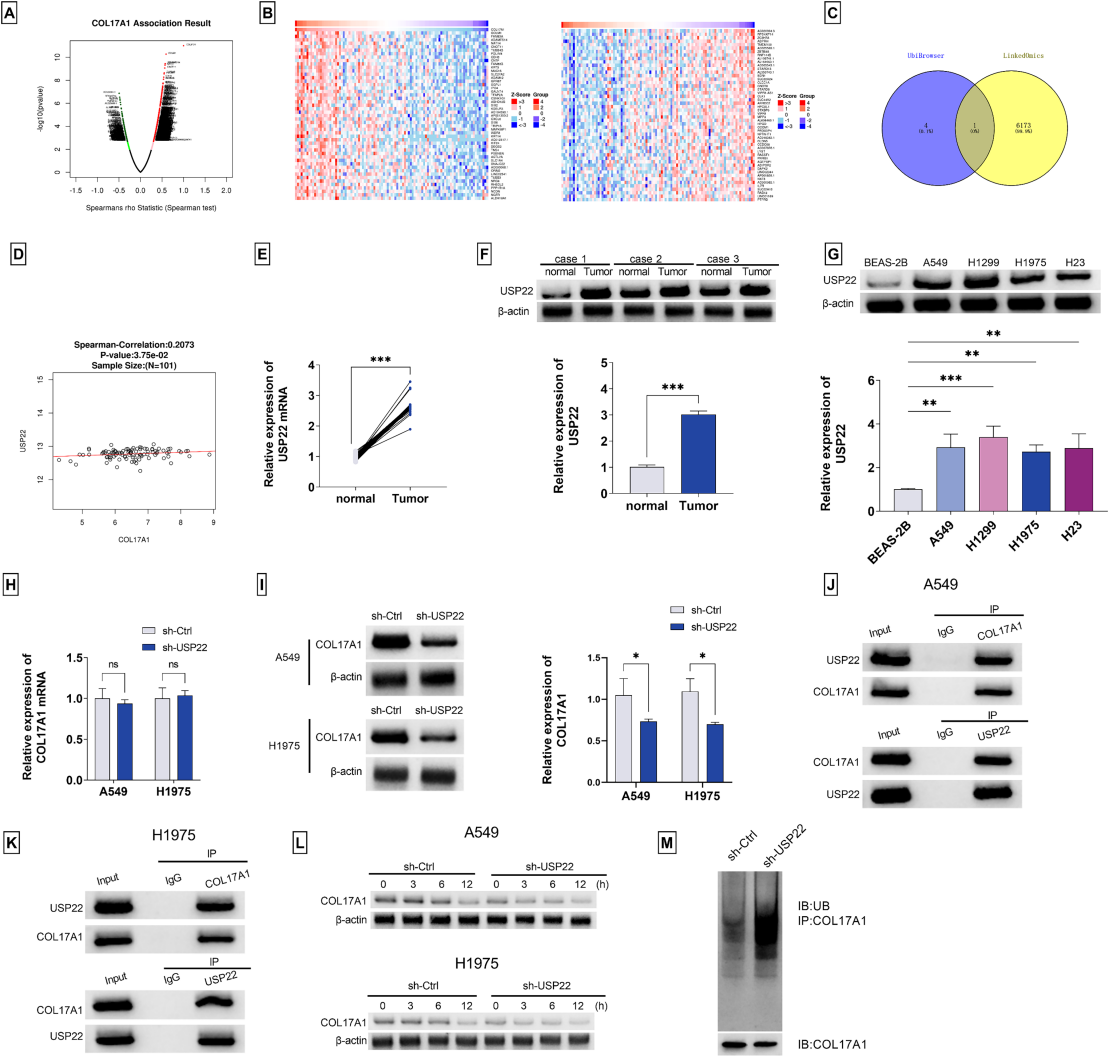
USP22 deubiquitinates and stabilizes COL17A1. (A) Volcano plots depicting the genes related to COL17A1 in LUAD using the LinkedOmics database. (B) Heat maps depicting the top 50 genes with the most significant positive association and the top 50 genes with the most significant negative association with COL17A1 in LUAD (LinkedOmics database, Pearson's correlation coefficient > 0.5, *p* < 0.05). (C) Venn diagram depicting the selection of USP22 by combining the 6174 genes with significant positive association of COL17A1 in LUAD (LinkedOmics database) with 5 COL17A1‐associating deubiquitinases (UbiBrowser2.0 database). (D) The positive correlation of USP22 with COL17A1 expression in LUAD dataset (LinkedOmics database). (E) Quantitative PCR for USP22 mRNA in a cohort of LUAD tumors (*n* = 45) and matched normal lung tissues (*n* = 45). (F) USP22 protein levels between LUAD tumors (*n* = 3) with matched normal lung tissues (*n* = 3) detected by immunoblot analysis. (G) USP22 protein levels in four human LUAD cell lines (A549, H1975, H1299, and H23) versus normal BEAS‐2B cells. Expression analyses of COL17A1 mRNA (H) and protein (I) in sh‐Ctrl‐ or sh‐USP22‐transfected A549 and H1975 LUAD cells. (J,K) Co‐IP experiments with lysates of A549 and H1975 LUAD cells using an antibody specific for COL17A1 or USP22, followed by immunoblot analysis for USP22 and COL17A1 enrichment levels. (L) Sh‐Ctrl‐ or sh‐USP22‐transfected A549 and H1975 LUAD cells were exposed to 20 ng/mL of cycloheximide for the indicated time frames, followed by immunoblot analysis for the COL17A1 protein level. (M) IP experiments with lysates of A549 cells using the anti‐COL17A1 antibody, followed by immunoblot analysis (IB) for the ubiquitinated COL17A1 protein level using an antibody against ubiquitin (UB). *p < 0.05, **p < 0.01, ***p < 0.001. ns: nonsignificant.

Protein stabilization by epigenetic deubiquitination mechanisms plays a critical role in a series of protumorigenic processes [10]. In order to elucidate the molecular basis of COL17A1 in affecting LUAD progression, we focused on the deubiquitination mechanisms underlying COL17A1 upregulation. Utilizing the LinkedOmics database, we observed thousands of genes related to COL17A1 in LUAD, as illustrated by volcano plots (Figure 5A). The heat maps depicted the top 50 genes with the most significant positive association and the top 50 genes with the most significant negative association with COL17A1 in the LUAD LinkedOmics database (Spearman's correlation coefficient > 0.5, *p* < 0.05) (Figure 5B). By combining the 6174 significant positive association genes with 5 COL17A1‐associating deubiquitinases (USP22, USP33, USP8, USP50, and USP9X) predicted by the UbiBrowser2.0 database, only USP22 was found (Figure 5C), which has been identified as an oncogenic driver in LUAD [21, 22]. The LinkedOmics database showed a positive expression correlation between USP22 and COL17A1 in the LUAD dataset (Figure 5D). Through quantitative PCR and immunoblot analyses, the upregulated expression of USP22 was confirmed in human LUAD tumors and cell lines (Figure 5E–G). Further, regulation of USP22 in COL17A1 was examined. Although the COL17A1 mRNA expression was not altered by sh‐USP22, which could effectively silence USP22 in LUAD cells (data no shown), the COL17A1 protein level was significantly inhibited by the reduced USP22 expression in A549 and H1975 LUAD cells (Figure 5H,I), indicating the positive modulation of USP22 in COL17A1 protein expression. Using Co‐IP experiments with an antibody specific for COL17A1 or USP22, we confirmed the binding relationship between COL17A1 and USP22 in A549 and H1975 LUAD cells (Figure 5J,K), suggesting the USP22/COL17A1 interaction in LUAD. Moreover, by blocking protein synthesis with cycloheximide, we found the decreased stability of the COL17A1 protein in USP22‐silenced A549 and H1975 LUAD cells (Figure 5L), demonstrating that the COL17A1 protein can be stabilized by USP22. More intriguingly, in A549 LUAD cells, immunoblot analysis with an antibody against ubiquitin (UB) after IP experiments using the anti‐COL17A1 antibody revealed that USP22 silencing resulted in a distinct increase in ubiquitinated COL17A1 protein level and COL17A1 degradation (Figure 5M), suggesting that USP22 induces the deubiquitination of the COL17A1 protein in LUAD cells. Together, these results suggest that USP22 stabilizes COL17A1 by deubiquitinating COL17A1.

### 3.6 Reexpression of COL17A1 Reverses USP22 Silencing‐Induced In Vitro Phenotype Changes of LUAD Cells

**FIGURE 6:**
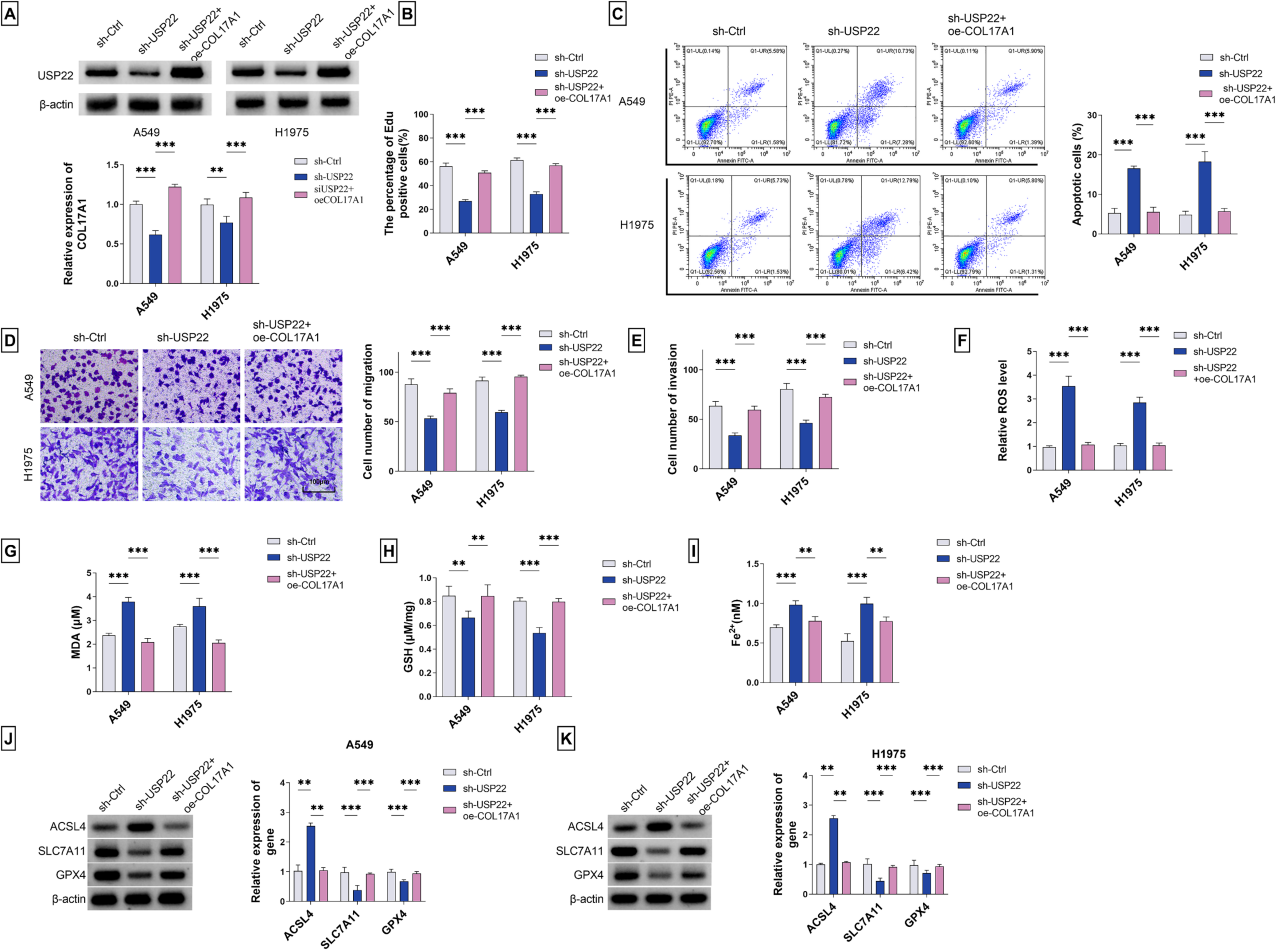
Regulation of the USP22/COL17A1 axis in LUAD cell in vitro proliferation, apoptosis, migration, invasion, and ferroptosis. (A) COL17A1 protein levels in A549 and H1975 LUAD cells after transfection of sh‐Ctrl, sh‐USP22, or sh‐USP22 + oe‐COL17A1 detected by immunoblot analysis. (B) Proliferation of A549 and H1975 LUAD cells after the indicated transfection assessed by EdU assay. (C) Apoptosis of A549 and H1975 LUAD cells after the indicated transfection evaluated by flow cytometry assay. Migratory (D) and invaded (E) capacities of A549 and H1975 LUAD cells after the indicated transfection measured by transwell assay. (F) Relative ROS content of A549 and H1975 LUAD cells after the indicated transfection measured by fluorescence assay. The levels of MDA (G), GSH (H), and Fe^2+^ (I) in A549 and H1975 LUAD cells after the indicated transfection detected by using the relevant assay kit. (J,K) The protein levels of ACSL4, SLC7A11, and GPX4 in A549 and H1975 LUAD cells after the indicated transfection. **p < 0.01, ***p < 0.001.

Since our data have demonstrated the regulation of USP22 in COL17A1 stability in LUAD cells, we next hypothesized that COL17A1 might mediate the functional effect of USP22 on LUAD progression. To resolve this question, we reintroduced COL17A1 with an engineered expression plasmid (oe‐COL17A1), which could effectively elevated COL17A1 protein expression in sh‐USP22‐transfected cells (Figure 6A). Remarkably, we found that sh‐USP22 transfection resulted in retarded proliferation and enhanced apoptosis of A549 and H1975 LUAD cells, which could be abolished by COL17A1 reexpression (Figure 6B,C). Similarly, USP22 silencing by sh‐USP22 transfection weakened cell migratory and invaded capacities, whereas reexpression of COL17A1 could rescue the retarded migration and invasion (Figure 6D,E). Furthermore, in A549 and H1975 LUAD cells, USP22 silencing induced a significant upregulation in ROS, Fe^2+^, and MDA contents as well as a clear reduction in GSH expression, while reexpression of COL17A1 had a counteracting impact on these changes induced by USP22 silencing (Figure 6F–I). Additionally, A549 and H1975 LUAD cells with USP22 silencing exhibited a higher expression of ACSL4 and lower levels of SLC7A11 and GPX4 than sh‐Ctrl controls; however, these changes could be markedly reversed by COL17A1 reexpression (Figure 6J,K), indicating that USP22 silencing induces cell ferroptosis via COL17A1 downregulation. Taking together, these findings demonstrate our hypothesis that the USP22/COL17A1 axis plays a crucial role in promoting LUAD progression.

## 4 Discussion

Collagen is an important component of the cancer microenvironment and has emerged as an essential modulator in tumorigenesis and malignant progression [23, 24]. Among these modulators, COL17A1 possesses critical activity in the early stages of cancer development [4]. Moreover, the crucial functions of COL17A1 in tumor progression have been established by a lot of documents. For example, COL17A1 is overexpressed in colorectal cancer and exerts oncogenic activity in disease malignant progression [6]. It was previously reported that hypomethylation of the *COL17A1* promoter occurs in LUAD [9]. However, the precise action of COL17A1 in LUAD progression is not well studied. In this paper, we demonstrate the upregulation of COL17A1 in human LUAD and its oncogenic activity in LUAD for the first time. Furthermore, our study demonstrates one novel deubiquitination mechanism driving COL17A1 upregulation.

Following three computational algorithms showing the high COL17A1 expression in LUAD, we confirm the upregulation of COL17A1 in human LUAD tumors and cell lines, implying its involvement in the pathogenesis of LUAD. Our loss‐of‐function experiments indicate that the disruption of COL17A1 in LUAD cells acts for the suppression of cell growth, invasion, and migration in vitro, as well as tumorigenicity in vivo. In addition, the disruption of COL17A1 can induce LUAD cell in vitro apoptosis. In recent years, the implications and crucial roles of ferroptosis in cancer biology have been highlighted [25], and intensive research is focusing on yielding novel approaches of harnessing ferroptosis for cancer therapy [26, 27]. In LUAD, ferroptosis resistance can contribute to disease progression [2]. By analyzing ferroptosis‐related factors, we demonstrate, for the first time, that COL17A1 KD induces ferroptosis in LUAD cells. Thus, we propose that COL17A1 is a potential oncoprotein in LUAD, and silencing COL17A1 may be an encouraging strategy for LUAD treatment.

The ubiquitination and deubiquitination of proteins, as well as other posttranscriptional modifications, can augment protein diversity and control protein fate. In cancer cells, protein deubiquitination induced by the deubiquitinase family can contribute to human carcinogenesis [28]. Recent reports have revealed that several deubiquitinases enhance lung tumorigenesis and therapy resistance by increasing the deubiquitination of certain oncogenic factors. For instance, USP5 deubiquitinates and stabilizes the immunosuppressive molecule PD‐L1, thereby promoting the progression of lung cancer [29]. USP9X enhances the deubiquitination of KDM4C to elevate radioresistance of lung cancer cells by activating the TGF‐β2/Smad signaling [12]. In this paper, when we investigated the deubiquitination mechanisms underlying COL17A1 upregulation, we found the deubiquitinase USP22. USP22 plays a complex role in carcinogenesis, immune escape, and tumor resistance to drugs [30]. For instance, USP22 can enhance the deubiquitination and stabilization of PD‐L1 in cancer cells, suggesting its protumorigenic role in cancer [31]. Conversely, USP22 has been demonstrated to exert an anticancer effect on colorectal cancer by diminishing mTOR activity [32]. These contradictory functions of USP22 may be due to the different cancer microenvironments. In LUAD, the upregulation of USP22 is positively associated with disease malignant progression and cisplatin resistance [33, 34]. The protumorigenic activity of USP22 in LUAD depends on its regulation in the expression of various oncoproteins, such as ALDH1A3 and SHH [22, 34]. In this paper, we establish the fact, for the first time, that USP22 stabilizes COL17A1 by deubiquitinating COL17A1 in LUAD cells, highlighting a novel deubiquitination mechanism underlying COL17A1 upregulation in LUAD. Our rescue analyses further demonstrate that the USP22/COL17A1 axis plays a crucial role in promoting LUAD progression in vitro. However, the exact functions of the new axis in LUAD progression in vivo are lacking, which will be warranted in future work. Exploring the ubiquitination site of COL17A1 and deubiquitination functional region of USP22 in regulating COL17A1 would be better and valuable for the function study of COL17A1 in LUAD. Such analyses are hampered at present by the lack of these investigations. Additionally, COL17A1 has been shown to modulate cancer phenotypes through the mTORC2 and AKT/mTOR pathways [7, 35]. Further uncovering the downstream molecular mechanisms of COL17A1 in LUAD is very essential for the research of COL17A1's role. Related studies will be conducted in future work. Given the significant roles of USP22 and COL17A1 in tumor immune response [5, 8, 30], future work is required to explore the precise functions of the USP22/COL17A1 axis in the immune response of LUAD.

In summary, our findings define the oncogenic activity of USP22‐stabilized COL17A1 in LUAD. We expect that USP22 and COL17A1 will be promising targets for the establishment of novel therapeutic ways against LUAD.

## Author Contributions

Guangxi Chen conducted the experiments and drafted the manuscript. Dandan Du prepared figures, collected and analyzed the data. Haihua Wang contributed the methodology, operated the software and edited the manuscript. Huifeng Li designed and supervised the study. All authors reviewed the manuscript.

## Conflicts of Interest

The authors declare no conflicts of interest.

## Data Availability

The data that support the findings of this study are available from the corresponding author upon reasonable request.
